# Dermatophilus congolensis

**DOI:** 10.3201/eid2808.212573

**Published:** 2022-08

**Authors:** Rüdiger D. Ollhoff, Fabio C. Pogliani, Fábio P. Sellera

**Affiliations:** Pontifícia Universidade Católica do Paraná, Curitiba, Brazil (R.D. Ollhoff);; Universidade de São Paulo, São Paulo, Brazil (F.P. Sellera, F.C. Pogliani);; Universidade Metropolitana de Santos, Santos, Brazil (F.P. Sellera)

**Keywords:** Dermatophilus congolensis, bacteria, actinomycete, dermatophilosis, exudative dermatitis, zoonosis, René Van Saceghem, Belgian Congo

## *Dermatophilus* *congolensis* [dur″mə-tof′ĭ-ləs con-gō-len′sis]

**Figure 1:**
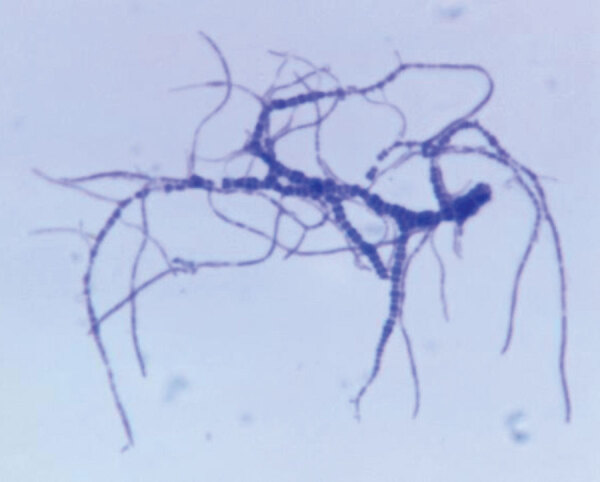
Photomicrograph of *Dermatophilus congolensis*, showing a Giemsa-stained, Gram-positive bacteria. Source: Dr. Jerrold Kaplan, Centers for Disease Control, 1965.

From the Greek *derma* (skin) + *philos* (loving), *Dermatophilus congolensis* is a Gram-positive, aerobic actinomycete, and facultatively anaerobic bacteria (Figure 1). *D. congolensis* infects the epidermis and produces exudative dermatitis termed dermatophilosis that was previously known as rain rot, rain scald, streptotrichosis, and mycotic dermatitis.

**Figure 2:**
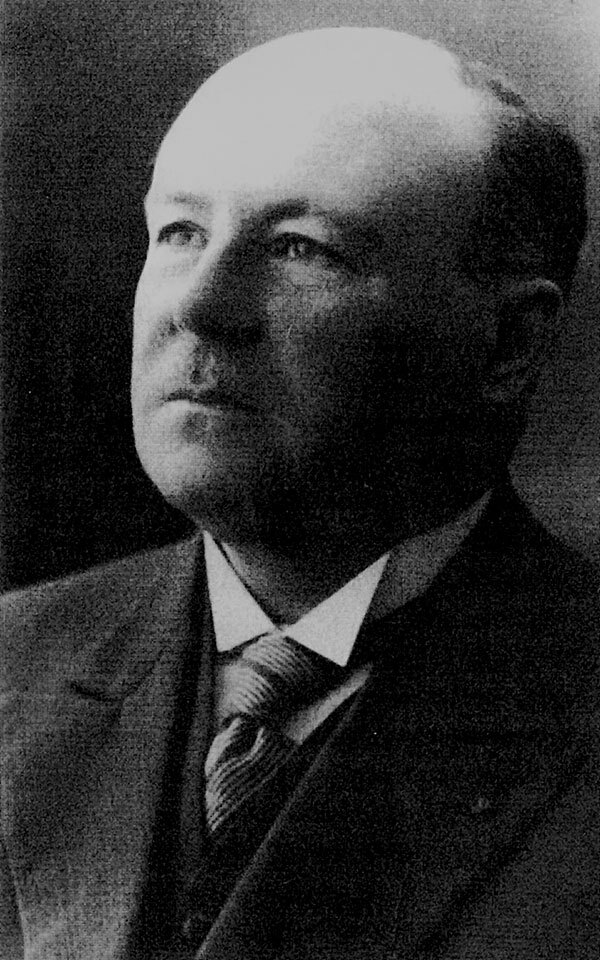
René Van Saceghem (1884–1965). Source: Mortelmans J. Veterinary medicine in Belgian Congo and Ruanda-Urundi from 1885 to 1962 [in French]. Vlaams Diergeneeskundig Tijdschrift. 2003;72:83–95. Courtesy of the Institute of Tropical Medicine (Antwerp). https://vdt.ugent.be/?q=nl/content/72-2-83-95

In 1915, René Van Saceghem (Figure 2), a Belgian military veterinarian stationed at a veterinary laboratory in the former Belgian Congo (thus, the species name *congolensis*), reported *D. congolensis* from exudative dermatitis in cattle. Local breeders and veterinarians had observed the disease since 1910, but the causal agent was not identified.

Dermatophilosis affects animals, mainly cattle, and more rarely humans. Outbreaks of *D. congolensis* infection have severe economic implications in the livestock and leather industries.
